# Neuromyelitis Optica Spectrum Disorder

**DOI:** 10.7759/cureus.48168

**Published:** 2023-11-02

**Authors:** Juliana Cazzaniga, Cesar E Jara Silva, Jonathan Quinonez, Samir Ruxmohan, Melissa M Leyva, Abrahim Fahs

**Affiliations:** 1 Neurology, Florida International University, Herbert Wertheim College of Medicine, Miami, USA; 2 Neurology, Larkin Community Hospital Palm Springs Campus, Hialeah, USA; 3 Addiction Medicine, Brandon Regional Hospital, Brandon, USA; 4 Neurocritical Care, University of Texas Southwestern Medical Center, Dallas, USA; 5 Osteopathic Medicine, Nova Southeastern University Dr. Kiran C. Patel College of Osteopathic Medicine, Fort Lauderdale, USA

**Keywords:** neuromyelitis optica spectrum disorder, brain anatomy, neurology and critical care, autoimmune neurological disease, wernicke's encephalopathy

## Abstract

Neuromyelitis optica spectrum disorder (NMOSD) is an autoimmune condition characterized by recurrent episodes of optic neuritis (ON) and transverse myelitis. This case report aims to highlight the importance of considering atypical presentations of NMOSD when confronted with MRI-detected Wernicke's encephalopathy. The primary target in NMOSD is the aquaporin-4 (AQP4) protein, predominantly located on astrocyte surfaces. Antibodies binding to AQP4 can lead to astrocyte dysfunction and damage, contributing to NMOSD's distinctive pathology. The associated immune response and inflammation can cause secondary harm to various components of the central nervous system, including oligodendrocytes and neuronal axons. This inflammatory process results in perivascular demyelination and axonal injury, further aggravating neurological deficits in NMOSD.

In this case, we present a 39-year-old female with no prior medical or surgical history who sought medical attention due to a three-week history of progressive eyelid heaviness and somnolence. NMOSD is an autoimmune condition primarily targeting the AQP4 protein, resulting in recurrent ON and transverse myelitis. The patient was initially misdiagnosed with myasthenia gravis due to somnolence and ptosis. Due to concerns about myasthenia gravis due to diffuse fatigue and bilateral ptosis, the patient was initially treated with intravenous immunoglobulin (IVIG) and admitted to the neurology service. On the first day of her hospitalization, MRI with and without contrast revealed extensive, non-enhancing T2-weighted-fluid-attenuated inversion recovery (T2-FLAIR) hyperintensities surrounding the third ventricle and affecting the periaqueductal grey, medial thalami, and mammillary bodies. There was also an interval increase in T2-FLAIR hyperintensity within the right medial temporal lobe, extending more posteriorly and inferiorly, abutting the temporal horn. Subsequent CSF encephalitis panel results showed positive West Nile virus (WNV) IgG but negative WNV IgM, and AQP4 antibodies were positive. Given the high specificity of AQP4 antibodies, the patient was diagnosed with neuromyelitis optica (NMO) encephalitis.

This case underscores the importance of considering atypical presentations of NMO when confronted with MRI-detected Wernicke's encephalopathy. Since our patient primarily displayed somnolence and eye-related symptoms, neither NMO nor Wernicke's encephalopathy were initially considered in the differential diagnosis. Furthermore, despite MRI findings suggestive of Wernicke's encephalopathy, it was considered less likely due to the absence of thiamine deficiency and consistent denials by family members regarding alcohol use, gastrointestinal issues, or inadequate oral intake. This case underscores the importance of considering NMOSD in patients with atypical symptoms, even when initial presentations suggest other conditions. Timely diagnosis is crucial to prevent mismanagement and improve patient outcomes. Clinicians should maintain a high level of suspicion for NMOSD, especially when MRI findings do not align with the initial diagnosis, as early recognition and treatment can significantly impact patient care and prognosis.

## Introduction

Neuromyelitis optica spectrum disorder (NMOSD) is an autoimmune condition characterized by recurrent episodes of optic neuritis (ON) and transverse myelitis [1-4]. Over 80% of NMOSD cases are attributed to IgG antibodies targeting aquaporin-4 (AQP4), a protein located on the surface of astrocyte cells. Antibody binding to AQP4 can result in astrocyte dysfunction and loss. Additionally, bystander inflammation may cause secondary damage to oligodendrocytes and neuronal axons, leading to perivascular demyelination in this disorder [5]. Common initial symptoms of NMOSD include ON, which manifests as blurred vision, reduced visual acuity, ocular pain during movement, and transverse myelitis with a range of sensory loss from mild to severe sensorimotor impairment in all four limbs, often accompanied by bowel and bladder dysfunction. Encephalopathy is a rare presentation in NMOSD, occurring more frequently in young children with MOG-IgG, where it can manifest as abnormal behavior, altered mental status, and seizures [6]. Encephalopathy is otherwise uncommon in adults with AQP4 NMOSD. Demyelination in other CNS regions can lead to various other presentations, such as intractable nausea, vomiting, and hiccups when the area postrema is affected, oculomotor disturbances and facial numbness with brainstem involvement, and hypothalamic and pituitary impairment, along with ataxia and coordination deficits when other diencephalic structures are involved.

Wernicke's encephalopathy is a well-documented complication of chronic vitamin B1 deficiency. This condition can manifest with symptoms of encephalopathy, oculomotor dysfunction (including nystagmus, lateral or conjugate gaze palsy), ataxia, and gait imbalance. However, the classic triad of symptoms is observed in only 16% of patients [7]. Imaging typically reveals hyperintensities in the bilateral medial thalami, the periaqueductal gray area, and the midbrain tectum on T2-weighted MRI [8]. The primary cause of Wernicke's encephalopathy is often a history of alcohol abuse, but it can also result from dietary deficiencies, absorption issues, prolonged vomiting, diarrhea, eating disorders, or chemotherapy [9].

Interestingly, patients with AQP4+ NMOSD have been observed to develop gastritis and vitamin B12 deficiencies [10]. NMOSD is also associated with other autoimmune disorders, including celiac disease, which can lead to absorption problems and vitamin deficiencies [11]. Furthermore, involvement of the area postrema in NMOSD can lead to persistent vomiting, contributing to nutritional deficiencies. There have been previous reports of NMOSD patients who mimic the symptoms of Wernicke's encephalopathy [12]. In this context, we present the case of a patient who presented with AQP4+ NMOSD alongside concurrent Wernicke's encephalopathy due to vitamin B1 deficiency.

This case report is unique for several reasons. Firstly, it highlights a rare presentation of NMOSD with concomitant Wernicke's encephalopathy. Such a dual diagnosis is exceptionally uncommon and underscores the importance of considering atypical neurological manifestations in patients with NMOSD. Additionally, the case emphasizes the need to broaden the differential diagnosis when patients present with symptoms of encephalopathy, oculomotor dysfunction, and altered mental status, as these could be attributed to conditions beyond the more typical etiologies. The aim of reporting this case is to shed light on the diagnostic challenges and complexities associated with NMOSD. Presenting a case where the initial presentation mimicked Wernicke's encephalopathy underscores the importance of considering autoimmune and demyelinating disorders in patients who do not fit classic clinical profiles. Furthermore, it highlights the significance of conducting a thorough clinical workup to reach the correct diagnosis, which is vital for appropriate treatment and management, ultimately improving patient outcomes. This case report serves as a reminder for healthcare professionals to maintain a high level of clinical suspicion, even in cases that present with symptoms that may initially seem unrelated to the primary condition.

## Case presentation

A 39-year-old female with no prior medical or surgical history was brought to the hospital by her family due to a three-week-long progressive experience of eyelid heaviness and sleepiness. The patient was unable to provide her medical history due to extreme drowsiness. However, according to her family, she had been in her usual state of health until approximately three weeks prior to her hospitalization. Initially, they observed unusual behaviors, including increased forgetfulness and severe fatigue, which led to her withdrawing socially and spending more time in bed. Over the following weeks, these symptoms worsened, with difficulty keeping her eyelids open and frequently falling asleep during meals, even leaving food in her mouth.

Upon her arrival at the hospital, the patient's physical examination revealed somnolence, bilateral ptosis, and restricted extraocular movements. Notably, she had limited abduction, upward gaze, and downward gaze, which were restricted to the mid-sclera, and bilateral absence of eye adduction. Additionally, there were no signs of an afferent pupillary defect or intranuclear ophthalmoplegia. Due to concerns about myasthenia gravis given the diffuse fatigue and bilateral ptosis, the patient was initially treated with intravenous immunoglobulin (IVIG) and admitted to the neurology service.

**Figure 1:**
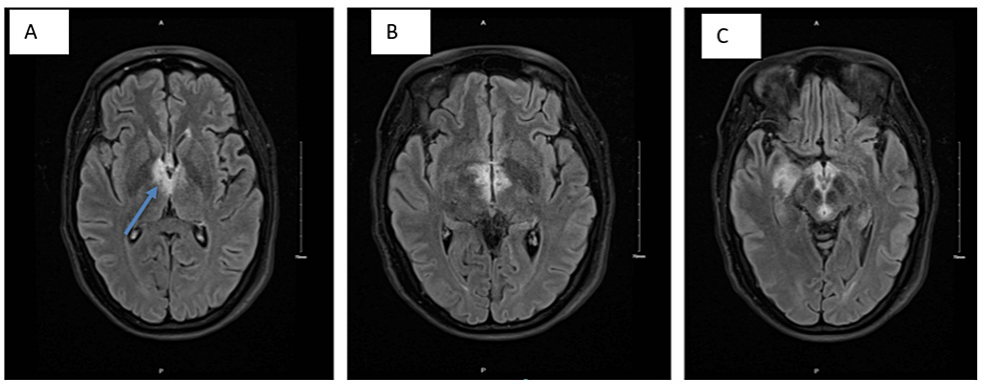
A, B, C: MRI brain T2-FLAIR axial view with and without IV contrast. (a) Hyperintensity around the third ventricle, with slightly increased prominence on the right. (b) Hyperintensity around the third ventricle, with slightly increased prominence on the right. (c) Hyperintensities involving the bilateral mamillary bodies, periaqueductal grey/tectal plate, around the third ventricle, anterior to the fourth ventricle, bilateral anteromedial thalami, right greater than left, interthalamic adhesion, right parahippocampal gyrus anterior to the right temporal horn

**Figure 2:**
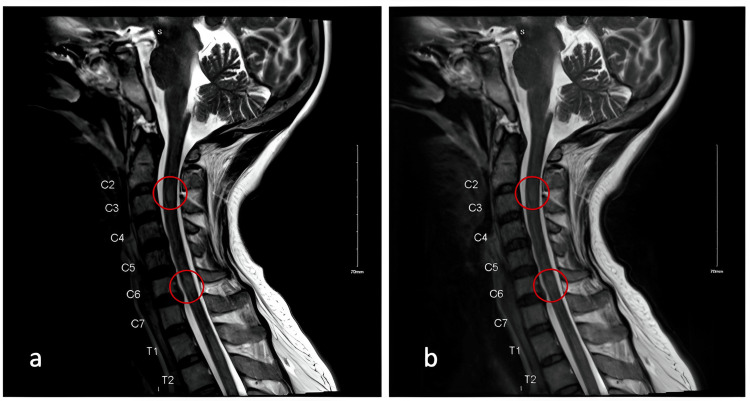
MRI T2-FLAIR cervical spine sagittal view with and without IV contrast. Moderate multilevel degenerative changes. Areas of T2-FLAIR hyperintensity posterior to C2 and C6 vertebral bodies (a, b)

**Figure 3:**
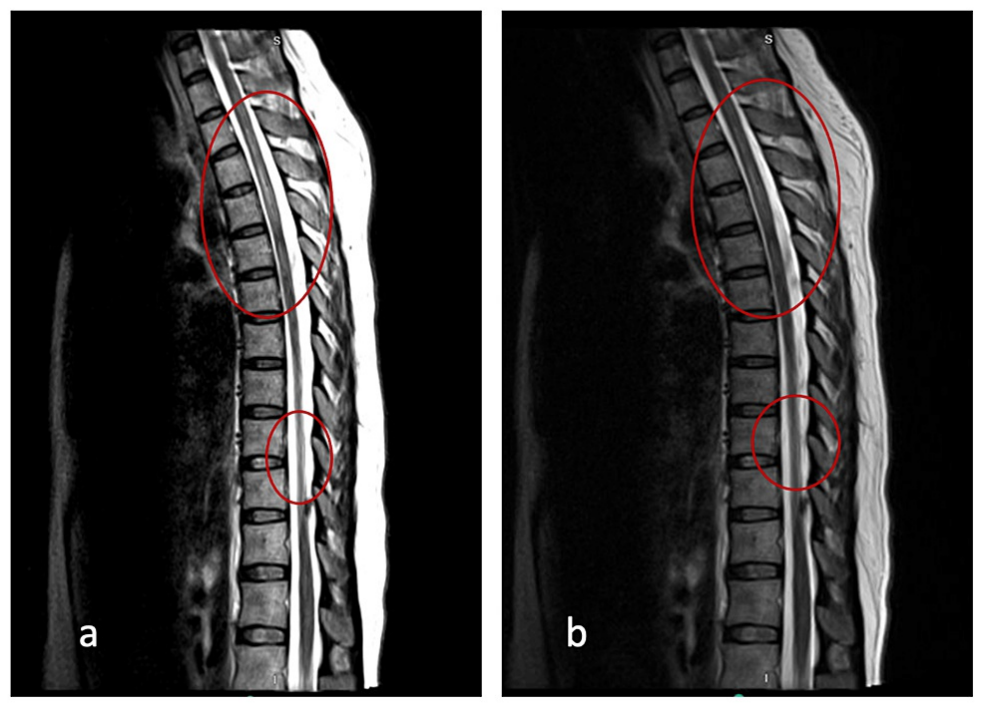
MRI T2-FLAIR thoracic spine sagittal view with and without IV contrast. Multiple patchy areas of abnormal signal scattered in the thoracic spine, most prominent at T1-T4, T5-T6, and T9 (a, b)

On the first day of her hospitalization, an MRI with and without contrast was performed, revealing extensive, non-enhancing T2-weighted-fluid-attenuated inversion recovery (T2-FLAIR) hyperintensities surrounding the third ventricle and affecting the periaqueductal gray, medial thalami, and mammillary bodies (see Figure 1A-1C). Similarly, hyperintensities can be seen in cervical (Figure 2) and thoracic MRI (Figure 3).

Initially, these imaging findings raised concerns about Wernicke's encephalopathy. However, family members who resided with the patient consistently denied any history of alcohol use or gastrointestinal surgery in her, affirming that she consumed regular meals. Consequently, the differential diagnosis was expanded to encompass arbovirus infection and demyelinating disease.

A thorough workup was initiated, including assessments of thiamine levels, MOG/NMO serologies, and comprehensive CSF studies for infectious, autoimmune, and paraneoplastic encephalitis, as seen in Table 1. Simultaneously, the patient was commenced on empiric thiamine and antiviral therapies. Despite these interventions, her condition continued to deteriorate over the subsequent days: her extraocular movements progressed to complete ophthalmoplegia, she developed recurrent hiccups, and her somnolence advanced to the extent that she necessitated intubation for airway protection. During this period, her workup revealed elevated serum thiamine levels, and initial CSF studies indicated the presence of 50 nucleated cells with a 95% predominance of lymphocytes.

**Table 1:** A summary of the patient’s labs The patient's CSF encephalitis panel returned positive for WNV IgG, although WNV IgM was negative, and AQP4 antibodies were positive. Because of the high specificity of AQP4 antibodies, that patient was diagnosed with NMO encephalitis NMO: neuromyelitis optica, AQP4: aquaporin-4, CSF: cerebrospinal fluid, IgG: immunoglobulin G, IgM: immunoglobulin M, WNV: West Nile virus

NMO/AQP4 FACS titer, CSF	1:20
CSF protein	83 mg/dL
CSF glucose	44 mg/dL
Appearance CSF	Clear
Color CSF	Colorless
RBC CSF	<1 cells/microL
Lymphs CSF	87 cells/microL
Mono macro CSF	5 cells/microL
Plasma cells CSF	8 cells/microL
Varicella zoster virus	Negative
WNV IgG	Positive
MOG	Negative
NMO/AQP4 FACS, CSF	Positive
AChR Ab, MuSK Ab serum	Negative
ANA	Positive
Syphilis​	Nonreactive

The patient underwent a combination therapy consisting of seven sessions of plasma exchange (PLEX) and five days of high-dose IV methylprednisolone treatment, followed by daily prednisone at 60 mg, with a planned taper during the transition phase with rituximab, which was delayed due to latent tuberculosis infection. However, due to some atypical MRI features for NMO, particularly the absence of enhancement in the brain and spine during an acute episode, along with the presence of a mesial temporal lobe lesion, a Whipple's test was conducted to rule out other conditions before initiating second-line Rituxan therapy following the initial combination therapy.

The patient's condition worsened with increased somnolence and respiratory decline during the first five PLEX therapy sessions and after completing her high-dose IV steroid treatment. However, there was a positive turn of events following her fifth PLEX treatment cycle, leading to the decision to extend her therapy with two additional treatments. During this period, her hospital course was further complicated by an ESBL urinary tract infection, urinary retention, hypotension, tachycardia, increased airway secretions, hypoxia, pneumonia, and poor nutrition, necessitating the placement of a percutaneous endoscopic gastrostomy (PEG) tube. Initiation of Rituxan was further delayed due to the need for treatment of latent tuberculosis infection. The patient remained highly somnolent, with periods of arousal lasting only a few minutes daily. Consultation with physical medicine and rehabilitation led to the recommendation to start methylphenidate to enhance these wakeful periods, allowing her to protect her airway better and participate in therapies. This ultimately enabled her to build up the strength and stamina needed to continue her recovery in inpatient rehabilitation after completing her initial Rituxan dose.

During her three-week stay in inpatient rehabilitation, the patient made significant progress. She continued to experience voiding issues, eventually requiring intermittent catheterization, but toward the end of her hospital stay, she regained the ability to void spontaneously. Swallowing difficulties persisted, leading to her being discharged with a PEG tube. Lastly, her issues with wakefulness prompted a change in medication to amantadine, with improvements observed during the last week of her hospitalization.

## Discussion

This case underscores the significance of considering atypical presentations of NMO when encountering MRI-detected Wernicke's encephalopathy. Initially, our patient's primary symptoms were somnolence and eye-related issues, which did not immediately lead to the consideration of either NMO or Wernicke's encephalopathy in the list of differential diagnoses. Notably, despite the MRI results aligning with Wernicke's encephalopathy, it was given lower priority in the differential diagnosis due to the absence of thiamine deficiency and consistent denials by family members regarding alcohol consumption, gastrointestinal problems, or poor dietary intake. Furthermore, even though the MRI findings were in line with those associated with NMO (including cervical and thoracic spine abnormalities and involvement of the area postrema) and ophthalmoplegia, NMO was also not initially considered due to the absence of imaging to assess optic nerve changes, a lack of active lesions in the brain and spine, and initial concerns about right medial temporal lobe findings, which were more suggestive of conditions like herpes simplex virus or atypical cerebritis [13].

The unique combination of NMO and Wernicke's encephalopathy makes this case interesting and distinctive from many previously reported NMO cases. One study found that experts frequently disagreed on the diagnosis of AQP4-Ab-negative NMO/MS overlapping patients [14]. The study also highlighted the difficulties in diagnosing NMO because some patients fulfilled the criteria while others did not but were still diagnosed [14]. Another case report found that a patient presented with ophthalmoplegia, cerebellar dysfunction, and altered mental state in addition to bilateral symmetrical periventricular lesions, which the original diagnosis was given as Wernicke's encephalopathy instead of NMO [15]. However, the case lacks the unique combination of NMO and Wernicke's encephalopathy [15]. While NMO often presents with recurrent episodes of ON and transverse myelitis, encephalopathy is a rare manifestation in adult patients with AQP4-IgG-positive NMO. In this regard, this case does not closely resemble typical NMO presentations, which makes it unique. However, there have been previous reports of NMO patients who mimic the symptoms of Wernicke's encephalopathy, suggesting some overlap between these conditions.

Regarding treatment, the case highlights the need for a multidisciplinary approach in NMO management. The use of PLEX and steroid therapy is consistent with clinical guidance for NMO treatment. However, the introduction of methylphenidate by the physical medicine and rehabilitation team to enhance wakefulness is an interesting addition to the treatment plan and showcases the adaptability required in complex cases. In terms of prognosis, the case report mentions a noticeable improvement in the patient's level of arousal with PLEX and steroid therapy. The use of PLEX is common in cases of severe NMO attacks. A typical PLEX cycle lasts two to three weeks and comprises five to seven cycles. However, this can vary as a meta-analysis by Queiroz et al. mentioned in clinical practice that cycles can range from 5 to 15 for treatment response [16,17].

The subsequent addition of methylphenidate further improved her prognosis. To make a comprehensive comparison with other NMO cases, it would be necessary to reference clinical studies and outcomes, especially in patients with similar atypical presentations and dual diagnoses. This would provide a more evidence-based perspective on prognosis and treatment outcomes. In summary, this case stands out due to its unique combination of NMO and Wernicke's encephalopathy, emphasizing the importance of considering atypical presentations and maintaining a multidisciplinary approach in NMO management. Further comparisons with similar cases and clinical studies would be valuable to enhance our understanding of the prognosis and treatment of such complex presentations.

In hindsight, it became evident that our patient's presenting symptoms were closely correlated with the areas most severely affected by the MRI. Her somnolence corresponded to the lesions in the periaqueductal grey, the hiccups were associated with lesions in the area postrema, and the ophthalmoplegia was linked to the extensive brainstem lesions. Ultimately, individuals presenting with a combination of somnolence, eye-related conditions, intractable nausea, vomiting, and hiccups, accompanied by MRI findings that match these clinical symptoms, should undergo a timely serological workup to ensure a prompt and accurate diagnosis.

## Conclusions

This case highlights the pivotal role of a multidisciplinary team comprising specialists in neurology, neurocritical care, and physical medicine and rehabilitation. The collaborative efforts of these disciplines led to complementary interventions that facilitated the patient's recovery. Once a diagnosis of NMO was established and the patient underwent PLEX and steroid therapy, there was a noticeable improvement in her level of arousal. Nevertheless, this improvement remained limited to a few brief periods each day. At this juncture, the physical medicine and rehabilitation team recommended the initiation of scheduled methylphenidate in the morning to enhance her wakefulness, a crucial turning point in her overall prognosis. In terms of prognosis, the case report mentions a noticeable improvement in the patient's level of arousal with PLEX and steroid therapy. The subsequent addition of methylphenidate further improved her prognosis.

In summary, this case stands out due to its unique combination of NMO and Wernicke's encephalopathy, emphasizing the importance of considering atypical presentations and maintaining a multidisciplinary approach in NMO management. Further comparisons with similar cases and clinical studies would be valuable to enhance our understanding of the prognosis and treatment of such complex presentations.
